# Some Things Training-Schools Have Done

**Published:** 1918-11-02

**Authors:** 


### THE MATRONS' AND SISTERS' DEPARTMENT.

### SOME THINGS TRAINING-SCHOOLS HAVE DONE.

Is there any justification for the confidence we have often expressed that the training-schools, now no longer isolated as in the past, but in full co-operation under the ægis of the College of Nursing, will be able to originate and carry through the reform of the nursing profession? It must be remembered that the reforms demanded are no mere stroke-of-the-pen alterations in the terms of engagement, education, and future prospects of the nurse. They involve widespread structural alterations to existing institutions and the intelligent reorganisation of vast undertakings dependent on public support, either through the grants of ratepayers or the free-will offerings of the benevolent. Can the training-schools move the springs of action in these public bodies? Can they ensure that money and brains will be forthcoming to give nurses what they desire to have? The answer lies in the history of nursing.

There are many among the earnest nursing reformers of to-day who can remember the type of Nurses' Home which was thought very well adapted to nurses' reasonable requirements in days gone by, even under Mrs. Wardroper of venerable memory at St. Thomas's. Narrow, often dark, cubicles containing bed, chair, and wash-stand; hurried meals of so unsatisfactory a character that it was taken for granted that every probationer must supplement her rations by surreptitious cups of tea with buns and pastries in her own quarters. One small crowded sitting-room. No slightest provision for recreation, for sick attendance, or study. Who could have believed that such Nurses' Homes, many unspeakably bad in ventilation and cleanliness, would ever give place to the finely planned and appointed buildings which are available for the staff in countless modern institutions? Had the spirit of revolt entered into the probationers of thirty or forty years ago, what could they have achieved! The modern Nurses' Home has come about on the initiative partly of far-sighted matrons, who could form an ideal, and partly of lay hospital authorities, convinced by the cogent arguments of reformers that in this direction lay advance for their institutions. But whether it were the Governors or the matron who first moved, assuredly no good Nurses' Home was ever built without the whole-hearted co-operation of all the authorities under the conviction that the prosperity of the training-school meant the prosperity of the entire institution, and that the training-school could never prosper unless the nurses were adequately housed. Direct pressure exerted by the probationers or by the permanent members of the staff has been an imperceptible element in the gradual process of revolution which has brought into being the modern Nurses' Home, and is still at work modifying, improving, introducing new features.

Money and brains, these are now, as formerly, the first requisites for reform. It is not merely that more money is required in the nursing department to raise salaries. It is that more nurses are required in every institution if better conditions are to be secured. More nurses mean larger Nurses' Homes, additional buildings at a period when for many years to come building will be more costly than ever before in this island. Better educational facilities mean more money for lectures, more money for scholarships, more money to attract women of higher education to the post of sister-tutor, more money for class-rooms, laboratory, library, models, and the like. None of these things can be had except on the initiative of the training-schools. In the hospitals in London, in the Provinces, and in Scotland the governors are keenly interested in the progress and prestige of their training-schools for nurses. The matrons who hold office do so as the result of their high reputation in training nurses and in maintaining the best traditions of the profession. The physicians and surgeons, whose reputation to some extent depends on the high quality of the nursing staff, demand the best brains which can be drawn into the Nursing Service. All the influences which are at work in the training-schools tend towards an improved status in the nursing staff. And this can only be brought about by the united counsels of the matrons and by financial support enlisted by the governing authorities. No longer is the matron compelled to think out her schemes alone, and suffer the bitter disappointment of seeing her cherished reforms postponed till her day is past. A swift momentum has been imparted by the establishment of the College of Nursing to the reforming movement which has been stirring so long. We await the future with confidence.

